# 3D-Printing Graphene Scaffolds for Bone Tissue Engineering

**DOI:** 10.3390/pharmaceutics14091834

**Published:** 2022-08-31

**Authors:** Amber F. MacDonald, Meaghan E. Harley-Troxell, Steven D. Newby, Madhu S. Dhar

**Affiliations:** College of Veterinary Medicine, University of Tennessee, Knoxville, TN 37996, USA

**Keywords:** graphene, nanoparticle, 3D printing, bone tissue engineering

## Abstract

Graphene-based materials have recently gained attention for regenerating various tissue defects including bone, nerve, cartilage, and muscle. Even though the potential of graphene-based biomaterials has been realized in tissue engineering, there are significantly many more studies reporting in vitro and in vivo data in bone tissue engineering. Graphene constructs have mainly been studied as two-dimensional (2D) substrates when biological organs are within a three-dimensional (3D) environment. Therefore, developing 3D graphene scaffolds is the next clinical standard, yet most have been fabricated as foams which limit control of consistent morphology and porosity. To overcome this issue, 3D-printing technology is revolutionizing tissue engineering, due to its speed, accuracy, reproducibility, and overall ability to personalize treatment whereby scaffolds are printed to the exact dimensions of a tissue defect. Even though various 3D-printing techniques are available, practical applications of 3D-printed graphene scaffolds are still limited. This can be attributed to variations associated with fabrication of graphene derivatives, leading to variations in cell response. This review summarizes selected works describing the different fabrication techniques for 3D scaffolds, the novelty of graphene materials, and the use of 3D-printed scaffolds of graphene-based nanoparticles for bone tissue engineering.

## 1. Introduction

There is a growing demand to engineer functional tissue using three-dimensional (3D) biological substitutes. Tissue engineering is a field composed of many scientific disciplines including biomedical engineering, cellular molecular biology, material science, and biochemistry. The concept of tissue engineering evolved in the 1990s whereby stem cells and materials could be implanted in vivo to restore injured tissues [1]. Since all tissues are derived from stem cells, conventional tissue engineering strategies have centered around stem cell-based therapies [2,3,4]. However, preparation of exogenous stem cells is a process that can take months—between isolation, expansion, characterization, and ensuring quality control (i.e., lack of viral contamination). Even then, stem-cell therapies are not FDA-approved and cause many concerns over regulation and safety [5,6,7]. Alternatively, scaffold materials that both support a defect and attract endogenous stem cells to the injured area is the future of tissue engineering [8,9,10]. Graphene materials have recently gained attraction for engineering new tissues [11,12,13,14]. However, most graphene studies have relied on two-dimensional (2D) surfaces, when native tissues are within a 3D environment [15,16,17,18,19,20,21]. Hence, the fabrication of novel biomaterials (including graphene derivatives) relies on 3D construction, which is feasible through many techniques, including 3D printing. In this review, we briefly discuss the different fabrication techniques for 3D scaffolds, the novelty of graphene materials, and their applications for 3D printing in tissue engineering, with a focus on bone regeneration.

## 2. Material Properties for Tissue Engineering

Although 3D printing is revolutionizing personalized treatment, the material needed to print the scaffold is a long-debated topic that depends on the desired tissue source to be repaired. These materials range anywhere from hydrogels, to nanoparticles, bio-metals, bio-ceramics, and bio-degradable polymers, each of which exhibit specific physicochemical properties. There are many material properties that influence tissue regeneration such as porosity, wettability, stiffness, strength, elasticity, biodegradability, and cytocompatibility. For example, a weight-bearing bone may require a stronger material that mimics the strength of the native tissue as the new bone regenerates, in comparison to a non-weight-bearing bone. Materials must withstand water absorption without rapid deterioration, yet gradually degrade overtime so that (1) new tissue can grow and function independently and (2) does not create a permanent implant. Additionally, many tissues require a 3D porous structure that allows blood vessel infiltration for constant nutrient transport as cells are building new tissue [22]. The optimal pore size may vary between different tissues, but typically ranges between 100–500 µm [23,24]. Thus, fabricating a porous structure is one variable that can be conveniently controlled by 3D-printing technology.

Finally, tissue engineering materials must demonstrate properties of cytocompatibility, including cell adherence, cell viability, and stimulation of cell differentiation. Studies have shown that 3D scaffolds support cytocompatibility better than their 2D control counterpart [25,26,27,28]. Overall, testing 3D-printed structures in vitro is a stronger predictor of tissue reconstruction outcomes before implanting in vivo. Since carbon nanomaterials are under study for treating multiple tissue defects, the remainder of this review will specifically focus on graphene materials and their future as a 3D-printed scaffold.

## 3. Carbon Nanomaterials

Carbon-based nanomaterials have gained attention for treating various tissue defects [29,30,31]. Nanomaterials refer to extremely small particles (generally 1–100 nm by dimension), yet are strong and light weight. Particles <100 nm Ø can enter cells, and those smaller than 40 nm Ø can enter the nucleus [32]. Intracellular components such as DNA, RNA, proteins, and lipids control the cell’s behavior and yet are very small nanometer structures. Therefore, nano-sized materials provide an attractive environment for optimal cell function. 

### Graphene Materials

Carbon nanomaterials include fullerenes, carbon nanotubes, nanodiamonds, carbon-based quantum dots, and graphene [33]. Of these, graphene is relatively the youngest and has rapidly emerged as a superstar due to its versatile properties in several industries from electronics to sporting equipment and medical science. Graphene comes from graphite, a gray crystalline mineral from rocks in South America, Asia, and North America. Graphite is easily recognized as the material within pencils, traditionally (but mistakenly) referred to as “pencil lead”. Graphite’s 3D structure contains millions of graphene layers that are weakly attached by van der Waals forces [34]. The carbon atoms are arranged as flat hexagonal rings, with each carbon covalently bonded to three other carbons. Despite its long-time existence, a graphene monolayer was not isolated until 2004 by Professor Sir Andre Geim and Professor Sir Kostya Novoselov, University of Manchester. Since then, graphene materials have been extensively studied in engineering several tissues including bone [35,36,37,38], cartilage [39,40,41], nerve [14,42,43], skin [44,45,46], and heart [47,48,49].

**Figure 1:**
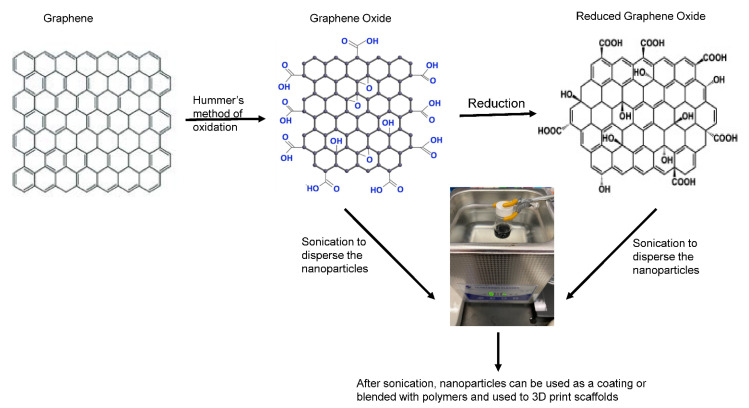
The structure of graphene, graphene oxide, and reduced graphene oxide. The color changes in the functional groups highlights the major changes in the chemical structure.

Pristine graphene, which is graphene in its original form, is hydrophobic (due to hydrocarbon contamination following air exposure) thereby lacks dispersion in water which raises aggregation/toxicity concerns when delivered in vivo [50]. This limitation has resulted in functionalizing graphene with hydrophilic groups that contain oxygen. Interestingly, this idea was discovered long before graphene was identified, when Benjamin Brodie oxidized graphite in 1859 [51]. Today, the most common method to oxidize graphite is by the Hummer’s method using a mixture of sulfuric acid, sodium nitrate, and potassium permanganate. Hereafter, graphite oxide layers are sonicated in water to exfoliate monolayers of graphene oxide (GO) (Figure 1). Unlike graphene, GO disperses in water and contains hydroxyl, carboxyl, and epoxy functional groups which allows it to be combined with other polymers or molecules for therapeutic use [52]. Typically, the C:O ratio in GO is 3 to 1 [53]. However, its exact composition can vary depending on the graphite source and the method of production. Therefore, the amount and distribution of oxygen functional groups may be similar, but not identical between GO sources [54].

Other functionalized graphene derivatives include reduced graphene oxide (rGO) which is an intermediate structure between graphene and GO, since it partially restores some properties lost during oxidation [55]. When GO is chemically reduced, some (but not all) of the oxygen functional groups are removed (Figure 1). In other words, rGO is the result of *reducing* the number of oxygen atoms found in GO. Reports estimate that rGO restores 80% sp^2^ structure with the remaining sp^3^ bonds derived from residual oxygen (C:O = 13:1) [56]. The reason for deoxygenation is because GO desensitizes the natural conductivity property of pristine graphene [57]. Therefore, rGO is favored for treating cardiac and neural defects as these tissues generate electrical signals.

## 4. Material Fabrication Techniques

**Figure 2:**
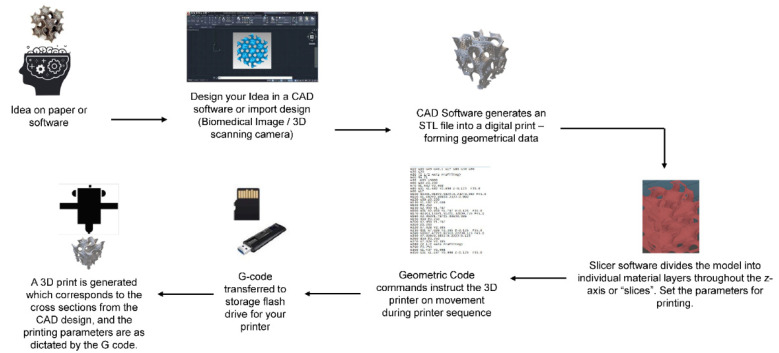
General scheme of a computer-controlled 3D-printing system.

There are several fabrication techniques to produce scaffolds which are categorized as either conventional or rapid prototyping (as summarized by Eltom et al., 2019) [58]. Conventional techniques include electrospinning, solvent casting, leaching, and phase separation [59,60,61]. With conventional techniques, however, there is poor control over architecture, pore network, and pore size, prompting challenges to consistently reproduce scaffolds with identical parameters [55,62]. On the other side, rapid prototyping uses computer software, more commonly known as computer-aided design (CAD), which designs scaffolds for production by a 3D-printing machine. Figure 2 describes the steps between software design and the final product of a 3D-printed scaffold. The design is first converted into a digital format using a Standard Tessellation Language (STL) file format. Using the STL file, the software ‘slices’ the design into multiple layers which are given values that denote how each layer is printed. Finally, a G-coding language is generated by the slicing software to communicate to the machine on how to move during printing. These files are then transferred to a 3D printer and the material of interest is subsequently printed into a 3D construct. A common 3D-printing technique is fused filament fabrication (FFF) whereby a thermoplastic polymer is melted above its glass transition temperature, extruded through the printer’s nozzle, and re-solidifies upon cooling on the print bed [56,63,64]. Other than the material extrusion method, there are several types of 3D printing techniques, including: vat photopolymerization processes (e.g., stereolithography), which create materials by exposing polymers to laser, light, or ultraviolet energy; binder jetting processes, which use a chemical bonding agent to fuse together powder particles; and powder bed fusion processes (e.g., selective laser sintering), which use thermal processes, such as electron beams or lasers, to fuse together powder particles. Each method has advantages and disadvantages beneficial for tissue engineering, depending on the polymer used and the desired outcome of the printed scaffold [65,66].

In tissue engineering, fabricating 3D-printed scaffolds has gained much popularity due to their speed, accuracy, reproducibility, and overall ability to personalize treatment whereby scaffolds are printed to the exact dimensions of a tissue defect. Most recently, there is new excitement of 3D printing directly into a patient’s body. For example, when diseased tissues are extracted during surgery, 3D printing technology could directly fill the open cavity for faster recovery and less pain post-surgery.

## 5. 3D Printing of Graphene Scaffolds

Many tissue engineering studies have fabricated graphene materials as a 2D cell culture substrate, with results indicating cell compatibility by enhancing gene/protein expression, proliferation, and differentiation [15,16,67,68]. However, a 2D cell culture substrate does not mimic the natural 3D tissue microenvironment. Developing 3D graphene scaffolds is the new standard, but most have been fabricated as foams which limit control of morphology such as the number of pores, the pore diameter, and the fiber diameter [69,70,71,72,73,74]. Table 1 summarizes the studies which have been successful in 3D printing a graphene construct for tissue engineering applications. The table lists the components of the graphene constructs used, along with the parameters used in printing. It is evident from the limited number of references listed in Table 1, that even though there are a number of publications using graphene nanomaterials, detailed information about the printing parameters is lacking. Therefore, despite the claim that it is necessary to 3D-print graphene scaffolds reproducibly with controlled properties, uniformity cannot be achieved.

**Figure 3:**
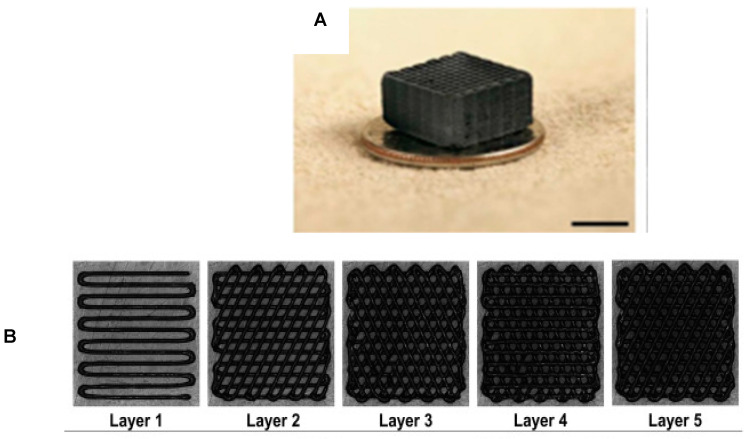
Images of 3D-printed graphene scaffolds. (**A**) Image of a 3D-printed GO aerogel with a microlattice architecture, adapted from [75]. (**B**) Images of each printed layer of a PCL-rGO scaffold, adapted from [55].

Zhu et al., 2015 was one of the first studies to successfully 3D-print a graphene construct with a microlattice architecture (as shown in Figure 3A) [75]. The intent of this study was to overcome the challenge of developing a printable graphene-based ink when maintaining its intrinsic properties (i.e., large surface area, stiffness, etc.). An ink gel was developed by combining a GO suspension with a silica filler which was loaded and extruded via the three-axis positioning stage (ABL 9000, Aerotech, Pittsburgh, PA, USA). The resulting construct was a porous GO aerogel with a cube-like structure. However, it should also be noted that aerogels are very low-density solids and easily collapse. Nonetheless, this study showed the future potential of 3D printing graphene materials with other polymers more suitable for tissue-engineering scaffolds. For example, Wei et al., 2015 printed rGO with thermoplastic polymers such as acrylonitrile-butadiene-styrene (ABS) or polylactic acid (PLA) [56]. rGO-ABS was prepared in concentrations of 0.4, 0.8, 1.6, 2.3, 3.8, 5.6, and 7.4 wt%. The majority of these concentrations extruded smoothly from the 3D printer (HOF1-X1, Nanjing Baoyan Automation Co., Ltd., Nanjing, China), but 7.4% rGO-ABS clogged the printer’s nozzle. It was noted however, that a more powerful homogenizing technique could allow more loading of rGO material. It was also recorded that the glass transition temperature (T*_g_*) of pure ABS alone was ~105.8 °C, which shifted to ~110 °C in presence of rGO. When printing any novel material, the correct Tg is necessary so that the material is softened (yet not melted) for extrusion and subsequent cooling at room temperature [79]. Finally, Jiang et al., 2018 successfully designed a porous GO hydrogel via 3D printing [76]. The ink was prepared by adding CaCl_2_ into a GO suspension whereby the Ca^2+^ ions could crosslink with the functional groups of GO to form a hydrogel. This method prevented any clogging within the nozzle, defied any collapsing, and maintained its shape upon printing. Overall, these studies were the first attempts to directly print a graphene material using a 3D-printing designed system.

More recently, Vijayavenkataraman et al., 2019 printed rGO scaffolds with the specific intent of engineering neural tissue [77]. rGO was mixed within polycaprolactone (PCL), but the exact concentration was unclear. Scaffolds were fabricated with the electrohydrodynamic jet (EHD-jet) printing system with an average fiber diameter of ~46 µm and pore size of ~125 µm consistent between both PCL and rGO-PCL scaffolds. As expected, the rGO-PCL scaffolds demonstrated better electrical conductivity (1.35 ± 0.3 mS/m) in comparison to its PCL control (0.09 ± 0.005 µS/cm). Interestingly, when PC12 cells were seeded, the rGO-PCL scaffolds stimulated more cell proliferation than PCL alone and supported expression of neural markers such as GAP43, β3-tubulin, and NF200. Overall, the data showed that rGO can be fabricated as a porous 3D scaffold, is cytocompatible, and should be further studied in vivo as a neural guide conduit.

Similarly, Seyedsalehi et al., 2020 mixed rGO within PCL at concentrations of either 0.5%, 1%, or 3% and successfully printed 3D scaffolds (strand size = 300 µm, pore size = 420 µm) with high consistency and repeatability (Figure 3B) [55]. Structures were printed using the 4th Generation 3D Bioplotter using parameters of: cartridge temperature (100 °C), platform temperature (10 °C), pressure (0.6 MPa), and speed (1.4 mm/s). Many material properties were examined including wettability, swelling, degradation, deformation behavior, compressive modulus, compressive strength, and cytocompatibility. After 14 days in simulated body fluid, it was found that PCL alone was hydrophobic, whereas the addition of rGO increased water uptake, swelling, and accelerated the rate of degradation. Interestingly, 0.5% rGO-PCL scaffolds had the best mechanical performance with compressive modulus and compressive strength enhanced by 150% and 185%, respectively. However, increasing rGO content to 1% and 3% deteriorated the mechanical performance, as the rGO sheets formed irreversible aggregates. Finally, all rGO concentrations had no adverse effects on human adipose-derived stem cells and supported cell viability in vitro. Overall, this study supported that combining small amounts of rGO within 3D-printed scaffolds reinforced biomechanical properties necessary for regenerating tissues and organs.

Interestingly, Nalesso et al. reported in vivo bone regeneration in critical sized defects using 3D printed scaffold of PCL and graphene nanoplatelets. In fact, the team fabricated electroactive scaffolds which enhanced bone regeneration in a rat calvarial bone defect model. Electroactivity of the scaffolds was modulated by varying the amount of graphene used [80]. Two recent reports of 2022 [81,82], further support that scaffolds consisting of graphene nanoparticles and commonly used polymers such as PLA, PCL, and 3D printed with specific parameters have the potential of being used in multiple tissue engineering processes. Gasparotto et al. used FFF 3D printing to develop two micropatterned scaffolds of PLA and graphene with 100 µm and 400 µm spacing between the filaments [81]. As expected, both the scaffolds were biocompatible but showed differences in cell behavior. The differences in the spacing lead to topographical differences, which resulted in alignment differences between neuronal, fibroblast and myoblast cells. Biscaia et al. used FDM techniques with a Bioextruder and analyzed the impact of processing conditions on the internal morphology of the scaffold filaments and their effects on the scaffold’s thermal, mechanical, and biological properties [82]. They further evaluated these properties under varying conditions of graphene. They used an immortalized and a primary mesenchymal stem cell culture to evaluate the biological properties of the scaffolds.

Alternatively, other laboratories have coated graphene onto 3D scaffolds to enhance mechanical strength and cytocompatibility [25,83]. For example, Li et al., 2020 first fabricated 3D-printed alginate (Alg) scaffolds before coating with rGO [25]. An Alg/Gel ink was printed using the 3D Bioplotter machine under parameters of room temperature, platform temperature (5 °C), speed (10 mm/s), strand spacing (1.5 mm), and extrusion air pressure (5 bar). Once printed, the Alg scaffolds were immersed in a GO solution until a uniform composition was achieved and thereafter reduced in ascorbic acid to ultimately produce a 3D rGO-Alg scaffold. Pore size varied from ~100–1000 µm due to multi-angled layers throughout the print. However, it is believed that various pore sizes are beneficial for tissue engineering as cell signaling is optimal at smaller pore sizes, and oxygen/nutrient transport is optimal at larger pore sizes [25,84,85]. Compared to Alg-only scaffolds, the coating of rGO increased the modulus by ~4 fold and demonstrated electrical conductivity. Interestingly, the proliferation of human adipose-derived stem cells on 3D rGO-Alg scaffolds was ~85% higher than cells grown on 2D rGO substrates. Additionally, expression of alkaline phosphatase (a bone mineralization marker) was five times greater on 3D rGO-Alg scaffolds than on 2D rGO substrates. Overall, the data support that rGO is supportive of cell attachment, proliferation, and osteogenic differentiation, and support the fact that 3D-printed scaffolds mimicking a natural tissue environment are important for tissue engineering.

## 6. Graphene and Bone Regeneration

It has thus, been established without a doubt that 3D construction of graphene scaffolds is the next step for clinical translation in tissue engineering. This research is important as the last decade of traditional 2D cell culture systems have shown that graphene substrates support stem cell differentiation into various lineages. These cell lineages are influenced by the concentration of graphene, its functionalization, shape, and the stem cell source [78,86]. But more specifically, multiple laboratories, including ours have found that graphene derivatives predominantly support bone differentiation [18,70,87,88,89,90,91,92,93,94,95,96,97,98,99,100,101]. A PubMed search using the phrase “graphene and bone” had more than double the publications of graphene and nerve, heart, muscle, and cartilage. Overall, the mechanical strength of graphene combined with its ability to support osteogenesis of stem cells, make it a forefront candidate in bone tissue engineering.

Although graphene materials have rapidly emerged as bone substitutes, few studies have examined the mechanisms behind its ability to induce osteogenesis. Some theories suggest the carbon arrangement imitates an organic bone ECM microenvironment, attracting cells to attach, self-renew, and differentiate [16]. An ECM microenvironment can be significantly affected by the topographic features of graphene substrates, thus, triggering variations in cell response. Spontaneous bone differentiation on 2D graphene substrates has been supported by calcium deposition and upregulation of bone-specific markers (i.e., ALPL, RUNX2, BMP2, SPP1, BGLAP, and COL I) [15,102,103]. These studies demonstrate the end result of osteogenic differentiation, but the underlying signaling pathways are still under investigation. It is difficult to reach a consensus because of the variations in cell source and graphene substrates used in these studies. For instance, Wei et al., 2017 found that bone marrow-derived mesenchymal stem cells (BM-MSCs) cultured on GO nanosheets had increased expression of β-catenin, thereby suggesting involvement of the Wnt/β-catenin pathway during osteogenic differentiation [103]. Xie et al., 2019 found that human dental pulp MSCs cultured on pristine graphene achieved osteogenesis via the integrin/focal adhesion kinase axis, thereby signaling SMAD phosphorylation, RUNX2 transcription, and production of SPP1 and BGLAP proteins [18]. Supportively, MacDonald et al., 2021 found that when human adipose-derived MSCs (AD-MSCs) and BM-MSCs were cultured on 6–10% oxygen containing graphene (LOG), multiple genes were involved during bone differentiation including genes related to cell adhesion, extracellular matrix, transcriptional regulation, BMP and SMAD signaling, growth factors, and angiogenic factors [15]. These results were also encouraging as stem-cell therapies derived from adipose tissue are much easier to obtain than stem cells derived from bone marrow. Therefore, any substrate material, such as graphene, that can nudge AD-MSCs into the bone lineage, is the preferred clinical strategy.

Despite this excitement, a major question is determining the best concentration and form of graphene that specifically sustains bone differentiation without collateral damage. BM-MSCs cultured on GO (0.1 µg/mL) had increased proliferation rates; however, at high GO concentrations (10 µg/mL), the BM-MSCs shrank and subsequently had reduced cell proliferation after just 3 days of culturing [104]. Similarly, Sun et al., 2021 found that silk fibroin/nanohydroxyapatite/GO (SF/nHA/GO) scaffolds loaded with urine-derived stem cells, had reduced osteogenic differentiation when GO concentrations exceeded 0.5% [105]. However, a different study found that 0.1% GO (combined with chitosan and hydroxyapatite), was an optimal concentration for cell adhesion, proliferation, and differentiation of MC3T3-E1 cells, a preosteoblast cell line [17]. In vivo, this concentration showed both osteogenic induction and no adverse reactions in a rat cranial defect model. Overall, before graphene is clinically applied as a bone biomaterial, it is very important to clearly understand the optimal concentration for all derivatives including pristine graphene, GO, and rGO. Additionally, the concentration could also change based on the stem-cell source, the shape and surface topography, and when the graphene source is combined with other polymers or drugs.

Despite this ongoing challenge, graphene materials have versatile ways in influencing bone regeneration. For example, graphene can indirectly support bone regeneration as a delivery vehicle that controls the release of potent BMP2 growth factors [102,103,106,107]. This helps to minimize the side effects of BMP2 reagents, but yet still provide a sustained stimulation of stem cells over time. GO was also used as a drug delivery platform to achieve a steady release of baicalin, a flavonoid compound widely used for both its osteoinductive and anti-inflammatory properties [108]. The surface area of graphene materials allows the immobilization of growth factors for targeted drug delivery that not only influences bone regeneration, but other tissues such as nerve and cartilage [40,86,109,110,111]. In other strategies, Hou et al., 2020 studied a 3D-printed graphene-PCL scaffold to conjunctively induce both cytotoxicity of Saos-2 cells (a human osteosarcoma cell line) and attract new bone regeneration. It was proposed that the gradual release of graphene could induce apoptosis of cancer cells, even as the remaining PCL layers provided the biomechanical environment to sustain the recruitment of healthy stem cells [90]. Overall, graphene materials have versatile properties for supporting bone regeneration including as a direct stimulator of new bone material, a delivery vehicle for other pharmaceutics, or targeting cancers of the bone.

## 7. Challenges in 3D Printing Graphene

**Figure 4:**
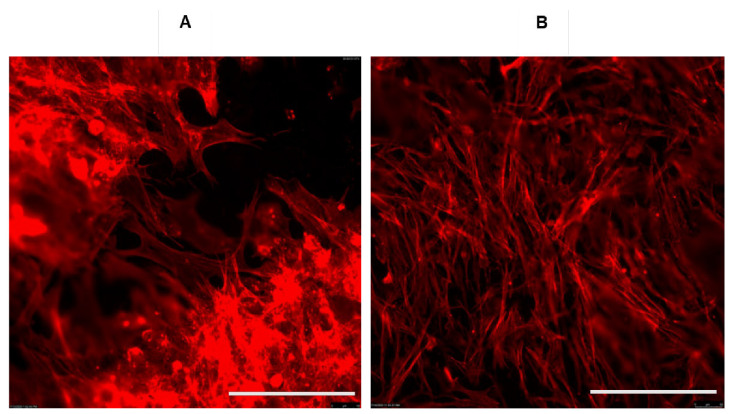
Cell response to rGO. Representative images to show a discrete pattern of cell adhesion and migration of human bone marrow-derived MSCs using Phalloidin 568 fluorophore. Phalloidin is a highly selective peptide which stains filamentous actin (F-actin) to demonstrate cytoskeletal organization of a cell when it adheres to a novel biomaterial. Note the changes in cell organization between day 1 (**A**) and day 21 (**B**), when MSCs adhere, proliferate and communicate in presence of graphene substrates. Scale bar = 50 μm.

Even though the potential of graphene and its derivatives in biomedicine has been realized, and, as described in the above section, 3D-printed scaffolds are important for the success of a bone tissue engineering strategy, practical applications of 3D-printed graphene scaffolds are still limited. Despite the availability of various 3D-printing techniques including fused deposition modeling, direct ink writing, selective laser sintering, and stereolithography, the use of 3D-printed scaffolds in human or veterinary medicine is limited. This can be attributed to variations associated with graphene manufacturing processes, leading to variations in cell response. For instance, the flaky texture of pristine graphene resembles sawdust particles, and thus is not a candidate for direct printing. In other words, the flaky texture draws challenges in creating pure graphene scaffolds via 3D-printing. This limitation is addressed by incorporating graphene flakes within a polymer (PLGA, PLLA or PCL) ink that sustains a 3D shape upon printing [112,113,114,115,116]. Additionally, pristine graphene can be derivatized for easy handling. Pristine or pure graphene is derivatized by oxidation or reduction processes. Two commonly used and commercially available graphene derivatives are GO and rGO. The two forms exhibit subtle variations in their physicochemical properties, leading to significant changes in cell behavior, which is of strong relevance to tissue engineering projects. According to a literature survey and the vendors’ specifications, the key difference between GO and rGO is their oxygen content. As stated in the previous section, GO contains a C:O ratio of 3:1, whereas rGO has a C:O ratio of approximately 13:1 [24,28]. The oxygen content indirectly affects the topographic features of GO and rGO, leading to changes in cell adhesion, proliferation and thus, differentiation (Dhar, unpublished). Recent data from our lab show varying effect on the osteogenic potential of rat and human adipose-derived MSCs when seeded on GO and rGO surfaces (Dhar, unpublished data). As a result, it is imperative that graphene derivatization is carried out using controlled conditions. It is also important to ensure that the mechanical process of 3D printing should not compromise the natural properties of graphene materials (i.e., strength, conductivity, surface area, etc.). Interestingly, we have also noted that the discrete cytoskeletal organization observed when cells adhere to graphene surface is an indicator of cell change. This can be visualized within 24 h after seeding cells on graphene in vitro. Monitoring changes in cell morphology over a period of time can not only demonstrate the cellular response to graphene, but can also be used to demonstrate changes in nanoparticles themselves. For instance, human bone marrow-derived MSCs show distinct changes in morphology and cell-to-cell communication in the presence of rGO (Figure 4). We are confident that these changes are in response to the topographic features of rGO because the substrates were fabricated under identical conditions (Newby and Dhar, Unpublished data). As a result, if a laboratory works with graphene-based nanoparticles, well established and validated in vitro and in vivo systems to test the graphene nanoparticles should exist. A multidisciplinary team science approach with coordinated efforts of material, analytical and biological scientists could be the key to the success of a 3D-printed graphene-based scaffold in biomedicine.

## 8. Toxicity Challenges of Graphene Materials

Despite the excitement of graphene materials and their use as a 3D scaffold for tissue engineering, their therapeutic use is still a novel idea and has yet to undergo a human clinical trial. As with any new substance, the primary question to address will always be safety. Since graphene nanoparticles exist in a variety of sizes (1 to 100 nm in length and 1–10 nm in thickness), and derivatives (oxidized or reduced with varying oxygen content), each form exhibits unique physicochemical properties, which can pose occupational and environmental risks to humans and animals. Information [117,118] regarding the toxicity of graphene is still uncertain, hence, additional investigations in biological systems (in vitro and in vivo) are required. Yet, medical research has raced to examine its physiological effects for various diseases, and simultaneously assessing and trying to improve its biocompatibility.

There are a growing number of in vivo reports regarding the toxicity of graphene materials [119,120,121,122,123,124,125,126]. An early study described by Yang et al., 2010 evaluated GO sheets coated and functionalized with polyethylene glycol (PEGylated nanographene sheets or NGS-PEG) in mouse tumor models [127]. After 40 days, systematic injection of NGS-PEG (20 mg/kg) specifically targeted the tumor site with no signs of toxicity or accumulation in the kidney, liver, heart, spleen, intestine, or lungs. Interestingly, when NGS-PEG was combined with photothermal therapy, the tumors were completely ablated, suggesting graphene’s potential for complementing current cancer treatments. However, it is important to note there was no control for NGS only, which may have had different toxicity outcomes, but also suggesting that a functionalized version of GO may help to overcome toxicity effects. The functionalization of graphene is not the only influential factor that may aid in overcoming toxicity effects. A review by Rhazouani et al., 2021 suggests that factors including method of administration and surface coatings can also influence how GO is excreted from the body [128]. The following studies provide evidence of how the variations in multiple factors influence systemic toxicity and how we can adjust these factors to improve biocompatibility.

Wang et al., 2011, evaluated GO toxicity in mice after 30 days of exposure to one of three concentrations: 0.1 mg, 0.25 mg, or 0.4 mg [129]. Results showed no mortality of mice exposed to 0.1 mg or 0.25 mg of GO. However, 4 of 9 mice died following GO injection at 0.4 mg. Histopathology results found GO conglomeration in the lung tissues, thus resulting in airway blockage and subsequent suffocation. When comparing lung tissues of all treatment groups, mice exhibited a dose-dependent series of granulomas after just 7 days of exposure. In other words, increasing GO concentration severely increased toxicity to the lungs. Overall, these results suggested that GO exposures could promote lung diseases.

A similar study from a separate laboratory also examined GO toxicity in mice following a low dose (1 mg/kg body weight) and a high dose (10 mg/kg body weight) via IV injection [130]. After 14 days, the 1 mg/kg dose of GO had no pathological changes in all organs tested (lungs, liver, spleen, and kidney). However, at 10 mg/kg, there was a high accumulation of GO in the lungs with pathological changes (i.e., granulomatous lesions, pulmonary edema, inflammatory cell infiltration, and fibrosis). The authors concluded that GO was biocompatible in most tissues, but higher dosages draw concern for abnormal changes within lung tissues.

With growing pulmonary toxicity concerns, Singh et al., 2012 compared the lungs of mice injected with either GO or graphene that was functionalized with amine groups (G-NH2) [131]. After 15 min, GO (250 µg/kg) stimulated vascular occlusion in lung tissue, although animals treated with G-NH2 (250 µg) had no signs of any occlusive pathology and instead demonstrated normal, healthy lung tissue. It was concluded that G-NH2 is not pro-thrombotic and is a safe graphene derivative, unlike other variations of graphene materials.

Schinwald et al., 2012 evaluated the risk of graphene nanoplatelets (GP) (average thickness of ~10 nm) following either inhalation or intrapleural injection in mice [132]. For inhalation, 50 µg of GP was added onto the tongue and held until at least 2 full breaths were completed. After 24 h, granulomatous lesions were present in the bronchiolar lumen of mice exposed to GP, but normal lung pathology was observed in both the vehicle and carbon control groups. Additionally, there was an increase in the total number of inflammatory cells (i.e., neutrophils, eosinophils) in the lavage fluid, and continued to show an inflammatory response one-week post-exposure. Secondly, an intrapleural injection of GP (5 µg) resulted in particle aggregations in pleural macrophages, indicating frustrated phagocytosis, an elevation of pro-inflammatory cytokine markers, and pleural thickening of the chest wall. Overall, the authors concluded that GP imposes a risk to the respiratory system, but acknowledged that the layer thickness is a key factor, and should be manufactured small enough that allows phagocytosis by macrophages.

Amrollahi-Sharifabadi et al., 2018 studied graphene oxide nanoplatelets (GONs) and how various doses measured using hematological and histological parameters after 21 days in vivo. They concluded that four cumulative doses of 50 (200 mg/kg) and 150 (600 mg/kg) did not show any toxicity effects in the serum levels, liver, kidney, spleen, lung, intestine, brain, or heart. However, 500 (2 g/kg) showed a significant change in all levels except the heart [133].

Most recently, Tabish et al., 2018 studied the toxicity of graphene nanopores (GNP) after a single IP injection or multiple injections (total of 14) over 27 days at doses of 5 mg/kg or 15 mg/kg in a rat model [32]. All doses (whether low or high, single or multiple) showed concerns in all tissues tested (liver, kidney, heart, small intestine, brain, testis, and lung), including tumor development within neural tissue of the brain. The pathological changes were presumably due to accumulation and low clearance of GNPs in the rat. In summary, the form of graphene, the dosage and the administration route, can all elicit an adverse effect in vivo. Toxicity can be alleviated by adjusting these factors. As a result, the proper dosage and administration route must be carefully examined before introduction of graphene materials in the clinic. More long-term in vivo studies are necessary to draw conclusions to minimize adverse effects of graphene materials.

## 9. Future Perspective and Conclusions

Conventional strategies of repairing tissue defects have relied on exogenous stem cells and 2D substrates. However, stem cell-based therapies have many limitations with future strategies turning to 3D structures that both support and attract cell differentiation within the injury site. Graphene, a carbon-based biomaterial, is under thorough research for repairing various tissues such as bone, cartilage, nerve, and heart. However, in vitro work of graphene has mainly been studied as a 2D monolayer or a 3D foam, whereby scaffold morphology is poorly controlled. With the revolution of 3D-printing technology, questions have asked whether graphene scaffolds can be 3D printed. Currently, there is a paucity of studies that have attempted 3D-printed graphene scaffolds for tissue engineering. These studies have mainly surfaced in the last few years, but it is expected that more developments will evolve in the future. Finally, 2D-graphene substrates have predominantly been studied in supporting new bone differentiation. Therefore, 3D-printed graphene scaffolds are the next step for clinical application in bone tissue engineering. However, understanding the optimal concentration of all graphene derivatives that balances both bone differentiation and minimizes toxicity is necessary prior to transplantation. Overall, there is great excitement over 3D-printed graphene scaffolds, but much work is necessary before standardization within tissue engineering.

## Figures and Tables

**Table 1:** **Summary of 3D-Printed Graphene Studies.** GO (graphene oxide); rGO (reduced graphene oxide); ABS (Acrylonitrile-butadiene-styrene); PLA (Polylactic acid); PCL (polycaprolactone).

Author, Year	Graphene Source	3D-Printer Model	Polymer	Printing Parameters	Overall Purpose
Zhu, 2015 [75]	GO	Silica	3-axis positioning stage (ABL 9000, Aerotech)	N/A	To demonstrate a 3D-printing strategy for graphene
Wei, 2015 [56]	rGO	ABS Or PLA	HOF1-X1	**rGO-ABS** Chamber Temp: 230 °C Platform Temp: 80 °C Nozzle: 130 °C Speed: 20 mm/s **rGO-PLA** Chamber Temp: 190 °C Platform Temp: 60 °C Nozzle:130 °C Speed: 20 mm/s	To demonstrate graphene is 3D printable
Jiang, 2018 [76]	GO	GO was crosslinked with Ca^2+^ ions to form a hydrogel	TH-206H	Room Temp Pressure: 2–3 bar Speed: 4–10 mm s^−1^	To enhance the functionality of 3D-printed graphene structures
Vijayavenkataraman, 2019 [77]	rGO	PCL	Electrohydrodynamic jet (EHD-jet)	N/A	To create a nerve guide conduit for neural regeneration
Seyedsalehi, 2020 [55]	rGO	PCL	4th Generation 3D Bioplotter	Temp: 100 °C Platform Temp: 10 °C Pressure: 0.6 MPa Speed: 1.4 mm/s	To evaluate printability, mechanical, and biological properties
Hou, 2020 [78]	Graphene	PCL	3DDisconvery^TM^ Evolution	Temp: 90 °C Screw Rotation Velocity: 8 rpm Deposit velocity: 12 mm/s Pressure: 6 bar	To create a scaffold for osteosarcoma and bone regeneration
